# Health Professionals of Prevention in Italy: The Value of Expertise During COVID-19 Pandemic

**DOI:** 10.3389/fpubh.2020.575500

**Published:** 2020-12-21

**Authors:** Malgorzata Wachocka, Fabio Pattavina, Vincenzo Palluzzi, Vito Cerabona, Patrizia Laurenti

**Affiliations:** ^1^Dipartimento Scienze della Vita e di Sanità Pubblica, Università Cattolica del Sacro Cuore, Rome, Italy; ^2^Fondazione Policlinico Universitario A. Gemelli Dipartimento di Scienze della Salute della Donna, del Bambino e di Sanità Pubblica, Rome, Italy; ^3^Dipartimento di Prevenzione, Azienda Sanitaria Locale Roma1, Rome, Italy

**Keywords:** prevention, COVID-19, occupational health, public health, health care professions

## Abstract

There are 22 different degree courses related to the Healthcare Professions in the Italian university system, which are divided into four areas. “Healthcare Professions of Prevention” is the fourth area and it is fundamental for the National Health Service. In particular, in this pandemic emergency situation, the contribution of the Prevention Technicians in the Environment and Workplaces (PTEW) is essential in the field and workplace management. The “Core Competence” of the PTEW is to carry out, with professional autonomy, prevention, verification, and control activities in the field of hygiene and safety of living and working environments. In the hospitals, the indications provided by national and/or regional authorities are implemented through procedures on good hygiene practices developed by PTEW (e.g., hand hygiene, “respiratory tract hygiene,” environmental hygiene, social distancing, and use of Personal Protective Equipment). One of the activities is the health surveillance on the field by population monitoring. The protocols foreseen for the “in-flow of workers” involve a wider control between social life and work. The PTEW will use a Check List divided into 3 macro phases: Entry, Activity Context, and Exit, defining each behavior of the work phases with a constant presence of verification of the procedures. The PTEW will be a Leader on the topics of education, training, and persuasion, considering a New Principle that “*transforms the worker as active part in the application and diffusion of the safety measures”*.

In the Italian university system the bachelor's degree related to the Health Professions is included uniquely in the Faculty of Medicine and Surgery. There are 22 different degree programmes that are divided into four areas of Health Professions^1^: Nursing and Obstetrics, Rehabilitation, Technicians and Prevention.

The common objective of these 22 courses is the health and the preservation of the population; this is particularly true if a study programme that involves a direct contact with the patient is chosen, but also taking into account the value of the Global Public Health^2^^,^^3^.

The Nursing and Obstetrics Health Professions involve a direct contact with patient. The Nurses are responsible for general nursing care and their main skills are disease prevention and care of patient. The midwives assist women during pregnancy, childbirth and puerperium, carry out deliveries and care for the newborn.

The area of the Rehabilitation Health Professions, which includes eight degree courses, aims to train highly specialized professionals in health rehabilitation, for example Physiotherapists, Speech Therapists, and Occupational Therapist, each with specific skills for the rehabilitation of the patient.

The area of Healthcare Technical Professions includes nine degree courses and trains technical figures specialized in different fields. These technicians work in two different areas, the technical-diagnostic area (Laboratory Technicians) and the technical-assistance area (Dental Hygienists) enabled to use the technical procedures necessary to perform disease diagnosis on biological materials or on the person.

The Health Care Professions of Prevention includes two degree courses to train professionals able to use prevention methodologies for the community or the individual health: Health Assistants (HA) and Prevention technicians in the environment and in the workplace (PTEW). In particular, the PTEW carry out, with professional autonomy, prevention, verification, and control activities in the field of environmental hygiene and safety in the workplace, food and beverage hygiene, public and veterinary health and hygiene^4^.

## Project/Program Methods

The “Core Competence” of the PTEW cover the activities that identify professional practice and “distinguish” it from other professions. Two essential elements explain the specificity of PTEW and are synergistic by enhancing this professional figure. The first element concerns the health specificity of the profile given by the professional mission that represents the guarantee of the citizen's health objectives. The second element is given by the wide exercise of the professional expertise that in an almost exclusive way, can be performed by the PTEW in the previously mentioned contexts. The definition and the regulations recognize the PTEW as a “health professional” who can practice both within the Public Prevention Departments and as a freelance profession^5^.

During this pandemic COVID-19 emergency in Italy, the contribution of the PTEW is fundamental hospital, territorial, and workplace management.

In the hospitals, the indications provided by national and/or regional authorities are implemented through procedures drawn up by PTEWs. Health and hygiene recommendations are implemented through education, training, promotion, and monitoring of good hygiene practices and field testing^6^ For example, through the promotion of hand hygiene, the promotion of adequate “respiratory hygiene,” environmental hygiene, social distancing and the use of appropriate Personal Protective Equipment (PPE). All workplaces represent, in fact, an important opportunity to spread the activity of sensitization on the correct behavior to be kept to reduce the transmission of the virus and the risk of contagion.

Regarding the Occupational Health in hospitals, the preventive measures to reduce the risk of infection in a workplace with COVID-19 disease are similar to those adopted for the general population. The related risk assessment document in the workplace must be updated in accordance with Legislative Decree no. 81/2008—transposition of EU Directive 89/391/EEC (81/08)^7^, which is the basis of Italian legal system in the matter of health and safety at work^8^.

The hospital employer has an obligation to provide his employees with correct information about the context in which we work. In according to 81/08, the employer must provide workers with the necessary and adequate PPE, the use of PPE and adequate awareness and training on how to use, dress, undress and disposal, are additional precautions necessary for healthcare workers in order to protect them and prevent the transmission of the virus in health and social environment.

In the COVID-19 situation, it is essential to pursue the objective of maximum possible protection of personnel, equipping them, on the base of evidence, with PPE of an appropriate level for the occupational risk they are exposed to.

In the field, the PTEW can work in multidisciplinary work teams in Prevention Departments within the Public Health Service (PHS), in the fight against the spread of Coronavirus by offering its professional contribution independently.

The health surveillance is implemented by population monitoring through daily contact to keep records of the symptoms attributable to Covid-19; in addition to the surveillance at home, the same activity takes place at the Extended Care, which currently are structures at greater risk of infection with the Coronavirus. The PTEW working in PHS have the task of managing mandatory notifications of infections from local hospitals, useful to epidemiological objectives.

The activities of the PTEW create networks and interconnections with the Stakeholders present on the field with the aim of promoting the Value of Population Health Prevention toward a culture of continuous improvement of the quality of care in terms of Safety and Patient Compliance.

The Coronavirus pandemic has upset and altered our habits by asking the Central Government to immediately introduce the Anti-Infection Protocols, fighting against Biological Risk, within the concept of “prevention.”

The Local Health Authorities, through the Prevention Departments, had to activate the new Protocols, for the risk related to Coronavirus, in every kind of company, facing a risk not specific to their activities and therefore without a training and culture about it.

The PTEW, will have to monitor the evolution of the Coronavirus related Risk without underestimated the other Risks: Chemical (use of sanitizing substances), Stress related work, Education, Training and Interferences (suppliers).

The protocols foreseen for the “in-flow of workers” will have a wider control between social life and work. The PTEW will intervene, in a more incisive way, using a Check List divided into 3 macro-phases: Entry, Context Activities, and Exit, defining each behavior in the different phases and sub-phases of work with a constant verification of the procedures.

The Occupational Safety and Health Administration (OSHA) through the “Guide to the preparation of workplaces for COVID-19” suggests to employers a useful reference for the correct management of the Covid-19 diffusion risk. With reference to the guideline, the HACCP concept could be introduced to analyse the hazards, critical control points (CCP) as used in the food safety^9^.

After the hazard analysis, the following CCP can be identified:

CCP1-ENTRY: Health status assessment (temperature), access route (CCP as subject to contamination). The disinfectant products, protective equipment and dressing procedures must be provided in the entrance area.

CCP2–CONTEXT ACTIVITIES: Consider the need to integrate emergency teams and reprogram emergency scenarios, supervise and correct protocols for precautionary measures (worker participation).

CCP3-EXIT: In high-risk workplaces (Covid patients) it is useful the use of existing decontamination techniques (e.g., asbestos sites).

## Discussion

The company's self-control plan with the support of controllers becomes indispensable in the management of Biological Risk by Covid-19.

The PTEW will be a Leader on Information, Training and Persuasion issues, considering a New Principle that “transforms the worker as active part in the application and diffusion of the safety measures” (1).

In the end “We don't have the luxury of applying urgent public measures to the society we want. We have to apply them to the society we have. That means that public health cannot offer magic bullets, as alluring as superhero status might be” (2).

## Data Availability Statement

The original contributions presented in the study are included in the article/supplementary material, further inquiries can be directed to the corresponding author/s.

## Author Contributions

MW, FP, VP, and VC had the idea and contributed to the writing of the text. PL carried out the final revision. All authors contributed to the article and approved the submitted version.

## Conflict of Interest

The authors declare that the research was conducted in the absence of any commercial or financial relationships that could be construed as a potential conflict of interest. The reviewer LJ declared a shared affiliation, though no other collaboration, with several of the authors MW, FP, and PL to the handling editor.

## Footnotes

^1^Decreto 3 novembre 1999, n.509 Regolamento recante norme concernenti l'autonomia didattica degli atenei.

^2^D.M. 19 febbraio 2009 Determinazione delle classi dei corsi di laurea per le professioni sanitarie, ai sensi del Decreto Ministeriale 22 ottobre 2004, n. 270.

^3^Decreto 22 ottobre 2004, n.270 Modifiche al regolamento recante norme concernenti l'autonomia didattica degli atenei, approvato con decreto del Ministro dell'università e della ricerca scientifica e tecnologica 3 novembre 1999, n. 509.

^4^Decreto Ministeriale 17 gennaio 1997, n. 58 Regolamento concernente la individuazione della figura e relativo profilo professionale del tecnico della prevenzione nell'ambiente e nei luoghi di lavoro.

^5^LEGGE 11 gennaio 2018, n. 3 Delega al Governo in materia di sperimentazione clinica di medicinali nonche' disposizioni per il riordino delle professioni sanitarie e per la dirigenza sanitaria del Ministero della salute.

^6^Rapporto ISS COVID-19 n. 4/2020 Rev. Indicazioni ad interim per la prevenzione e il controllo dell'infezione da SARS-COV-2 in strutture residenziali sociosanitarie.

^7^Decreto Legislativo 9 aprile 2008n n.81 e s.m.i. Testo unico sulla salute e sicurezza sul lavoro.

^8^Legge 23 dicembre 1978, n. 833, Istituzione del servizio sanitario nazionale.

^9^Guidance on Preparing Workplaces for COVID-19 U.S. Department of Labor Occupational Safety and Health Administration OSHA 3990-03 2020.
